# Dose advantage of abdominal deep inspiratory breath-hold (aDIBH) in postoperative adjuvant radiotherapy for left breast cancer

**DOI:** 10.1186/s43046-024-00234-2

**Published:** 2024-09-23

**Authors:** Junming Lai, Hui Luo, Shuang Hu, Fangyan Zhong, Rui Chen, Hong Lin

**Affiliations:** 1 Department of Oncology, GanJiang New Area Hospitalof, First Affiliated Hospital of NanChang University, NanChang, Jiangxi 330117 People’s Republic of China; 2 Department of Oncology, the First Affiliated Hospital of NanChang University, NanChang, Jiangxi 330006 People’s Republic of China; 3 Department of Acute Infectious Diseases, Third People’s Hospital of Jiujiang City, JiuJiang Hospital of Nanchang Medical College, Jiujiang, Jiangxi 332000 People’s Republic of China; 4 Jiangxi Medicine College, Nanchang University, NanChang, Jiangxi 330006 People’s Republic of China; 5 Department of Oncology, Jiujiang NO.1 People’s Hospital, Jiujiang Water of Life Hospital, Jiujiang, Jiangxi 332000 People’s Republic of China

**Keywords:** Breast cancer, Abdominal deep inspiration breath-hold, Radiotherapy, Dosimetry

## Abstract

**Purpose:**

We explored the dosimetric efficacy of the abdominal deep inspiration breath hold (aDIBH) technique using an audio-guided device in patients with left breast cancer undergoing postoperative adjuvant radiotherapy compared to free breathing (FB).

**Methods:**

A total of 35 patients with early stage left breast cancer underwent two computed tomography simulation scans each with aDIBH and FB after breast-conserving surgery. Treatment planning was optimized using the Pinnacle^3^ 9.10 planning system. The heart, left anterior descending coronary artery (LADCA), and left lung was defined as organs at risk (OARs). The dosimetric differences in the planning target volume (PTV) and OARs were compared between aDIBH and FB.

**Results:**

Compared with FB, the heart moved farther caudally and away from the chest wall, and the volume of heart became smaller under aDIBH due to expansion of the lungs. The D mean of the heart, LADCA and left lung of aDIBH were respectively reduced by 332.79 ± 264.61 cGy (*P* < 0.001), 1290.37 ± 612.09 cGy (*P* < 0.047) and 69.94 ± 117.73 cGy (*P* < 0.001). The V20 and V30 of the OARs were also significantly reduced with statistical differences (*P* < 0.05). In addition, there was no significant difference in the dosimetric parameters of the PTV between the two groups (*P* > 0.05).

**Conclusions:**

Implementation of the aDIBH technique for postoperative radiotherapy after breast-conserving surgery of the left breast cancer could reduce irradiation of the heart dose, LADCA dose and left lung dose, without compromising target coverage.

## Background

Breast cancer is the most common malignant tumor in women [1]. The International Agency for Research on cancer (IARC) of the World Health Organization released the latest global cancer burden data in 2020. Breast cancer has replaced lung cancer as the most common malignant tumor in the world. Postoperative radiotherapy is an indispensable component of the comprehensive treatment of breast cancer. Most patients receive whole-breast radiotherapy after breast-conserving surgery for early breast cancer. It has been proven to significantly reduce the risk of local recurrence and improve long-term survival in breast cancer patients [2, 3]. However, exposure of the heart and lungs to radiation can lead to radiation-induced cardiac and pulmonary complications, such as radiation-induced heart disease (RIHD) and radiation pneumonitis (RP) [4–6].

In recent years, photon beam radiotherapy based on linear accelerators has developed rapidly, such as three-dimensional conformal radiotherapy (3D-CRT), intensity-modulated radiotherapy (IMRT), and volume of rotating intensity modulated radiotherapy (VMRT), which allow highly complex radiotherapy treatment plans can be performed. However, the above techniques still cannot reduce the error of the target volume by the respiratory movement. The deep inspiration breath-hold (DIBH) technique can solve the respiratory-induced target motion during both simulation and treatment.

The DIBH technique involves the patient inspiring to a specified threshold and then holding that level of inspiration during radiotherapy. Compared with free breathing (FB), DIBH can move the heart further away from the chest wall, especially below the nipple level, and increase the volume of lung.

The DIBH technique can be divided into two types according to thoracic and abdominal breathing, thoracic DIBH (tDIBH) and abdominal DIBH (aDIBH). tDIBH is the process of deep inspiration and breath-holding mainly through the movement of the pectoralis and diaphragm during inspiration, and aDIBH is the process of deep inspiration and breath-holding mainly through abdominal muscle movement during inspiration. Moreover, a previous study revealed that aDIBH has more advantages in reducing the irradiation dose to the heart and lung compared with tDIBH and FB [7]. According to the implementation method, DIBH can be further divided into a voluntary deep inspiration breath hold (vDIBH) and a machine-initiated DIBH. The machine-initiated breath-hold includes the active breathing coordinator (ABC), real-time position management (RPM) [8] and the optical surface management system (OSMS) [9]. The HeartSpare multicenter clinical trials [10–12] conducted in the UK proposed the vDIBH technique, which did not require additional equipment assistance (such as ABC, RPM and OSMS.). vDIBH and ABC_DIBH were comparable in terms of positional reproducibility and normal tissue sparing. vDIBH was preferred by patients and radiographers, taken less time to deliver, and was cheaper than ABC_DIBH.

The aim of the present study was to evaluate the application of radiotherapy in aDIBH using an audio-guided device under active breathing training in postoperative adjuvant radiotherapy for left breast cancer, and to compare the dosimetric parameters with those of FB.

## Methods

### Patients

All included patients were left early breast cancer after breast-conserving surgery without distant metastasis, KPS ≥ 80 points, and normal cardiopulmonary function. The included patients agreed to receive CT scans of both aDIBH and FB breathing modes simultaneously. The included patients could cooperate with aDIBH using the abdominal muscles during inspiration for 20 to 30 s before the CT simulation.

### Breathing training

The radiation oncologist performed a training of vDIBH before CT simulation. The specific steps were as follows: the patient lay flat and breathed normally, took a deep breath with the chest remaining stable as much as possible, and made the upper abdomen bulge (abdominal breathing), held the breath for 20 to 30 s, returned to normal breathing, and repeated the above steps and training repeatedly to meet the requirements.

### CT simulation

The patients took off the coat and lay down in the middle of the AIO board with arms above their head and hands held in a grip (left hand down and right hand up) in the supine position, and a B pillow was placed under their head. The patients were placed on an integrated frame with a disposable thermoplastic body that served as a fixation device for setting reproducibility and inhibiting chest movement. To allow distension of the abdomen during aDIBH, a portion of the thermoplastic body covering the anterior abdominal wall and left breast was cut off by the radiation therapist. All patients underwent computed tomography (CT) simulation using a Siemens large-aperture CT simulator. The scan range was from the mandible to 3–5 cm below the mammary fold and the slice thickness was 5 mm. For each patient, CT scans of the DIBH and FB were acquired. For the CT scan of DIBH, the radiation therapists used an audio-guided device which was a simple broadcaster that can be connected to a computer, and the voice of the audio-guided device: take a deep breath, and then hold your breath. When patients underwent CT scans or treatments, radiation therapist used the audio-guided device to remind the patient to breathe deeply and hold breath according to the training state, and then conducted the scan and informed the patient could breathe normally after the scan. For the CT scan of the FB, the radiation therapists told the patient to breathe smoothly and freely and then conducted the scan.

### Volume delineation

The clinical target volume (CTV) and organs at risk (OARs) were delineated by the same radiation oncologist in all scans of FB and DIBH in the Pinnacle^3^ 9.10 planning system according to the Radiation Therapy Oncology Group (RTOG) Breast Contouring Atlas. The range of CTV was defined as follows: the upper boundary was the upper boundary of the breast or the second rib; the lower boundary was the lower boundary of the breast or the CT image of the contralateral breast disappeared; the internal boundary was the junction of the ribs and sternum; the outside was the outside of the breast or axillary midline; the anterior boundary was 5 mm below the skin including adipose tissue; and the posterior border was in front of the ribs excluding the ribs and pectoralis. For lymph node positive patients, CTV also includes the supraclavicular fossa. The planning target volume (PTV) was expanded by 5 mm based on the CTV. The upper boundary of the heart was at the level of the lower margin of the right pulmonary artery trunk and the lower boundary was at the apex of the heart. The entire length of LADCA was delineated down to the apical level excluding the left main trunk. The left lung was contoured to the entire left lung from the tip to the bottom, excluding mediastinal structures such as the hilus pulmonis, trachea, and bronchus.

### Treatment planning

Treatment planning was performed using the Pinnacle3 9.10 (Philips, Andover, MA, USA). Six to eight main tangential conformal fields were used with the field-in-field technique. Each field was divided into several sections so that the patients holding the breath for 20 to 30 s could complete the treatment. Irradiation mode was IMRT. The prescription dose was 5000 cGy in 25 fractions for all patients using 6 MV photon. Additionally, a boost to the tumor bed was recommended in patients at a higher risk of recurrence, and typical boost doses were 1000–1600 cGy in 5–8 fractions. All dose schedules were administered 5 days per week. Treatment planning had to meet the criterion that at least 95% of the PTV received 100% of the prescribed dosage and the dose of OARs should be kept as low as possible without compromising the PTV dose.

### Dosimetric parameters

Dosimetric parameters of PTV under FB and DIBH: volume size, mean dose (D mean), maximum dose (D max), homogeneity index (HI) [13] = ratio of the maximum dose (MD) divided by the prescription dose (PD), conformity index (CI) [13] = ratio of the prescription isodose volume (PI) divided by the target volume (TV). Dosimetric parameters of OARs (the heart, LADCA and left lung) under FB and DIBH: volume size, D mean and percentage of the organ volume receiving at least 5 Gy (V5), 10 Gy (V10), 20 Gy (V20) and 30 Gy (V30).

### Statistical analysis

Statistical analysis was performed using SPSS 22.0. The 2-sided Wilcoxon signed rank test was conducted for above dosimetric parameters. All values were reported as mean ± standard deviation (SD). *P* ≤ 0.05 was considered statistically significant. To ensure the accuracy of the data, the results were checked by two researchers. Additionally, a change in the dose-volume histogram (DVH) between the aDIBH and FB was designed.

## Results

### General characteristics

A total of 35 patients with early left breast cancer after breast-conserving surgery were enrolled in this study. 5 patients withdrew from this study due to uncooperative breathing training, and finally 30 patients (85.71%) were included. The median age of the included patients was 51 years (range, 30–66 years). Regarding the location of the mass in the breast quadrant, there were 19 cases in the upper outer quadrant, 5 cases in the upper inner quadrant, 4 cases in the lower inner quadrant, and 2 cases in the lower outer quadrant. All pathological types were invasive ductal cancer. According to the 8th edition of the American Joint Committee on Cancer (AJCC) staging system for breast cancer, there were 13 cases of pT1N0M0 (stage IA), 8 cases of pT2N0M0 (stage IIA), 4 cases of pT1N1M0 (stage IIA), 2 cases of pT2N1M0 (stage IIB), and 3 cases of pTisN0M0 (stage 0).

### Comparison of geometry and DVH

**Fig. 1:**
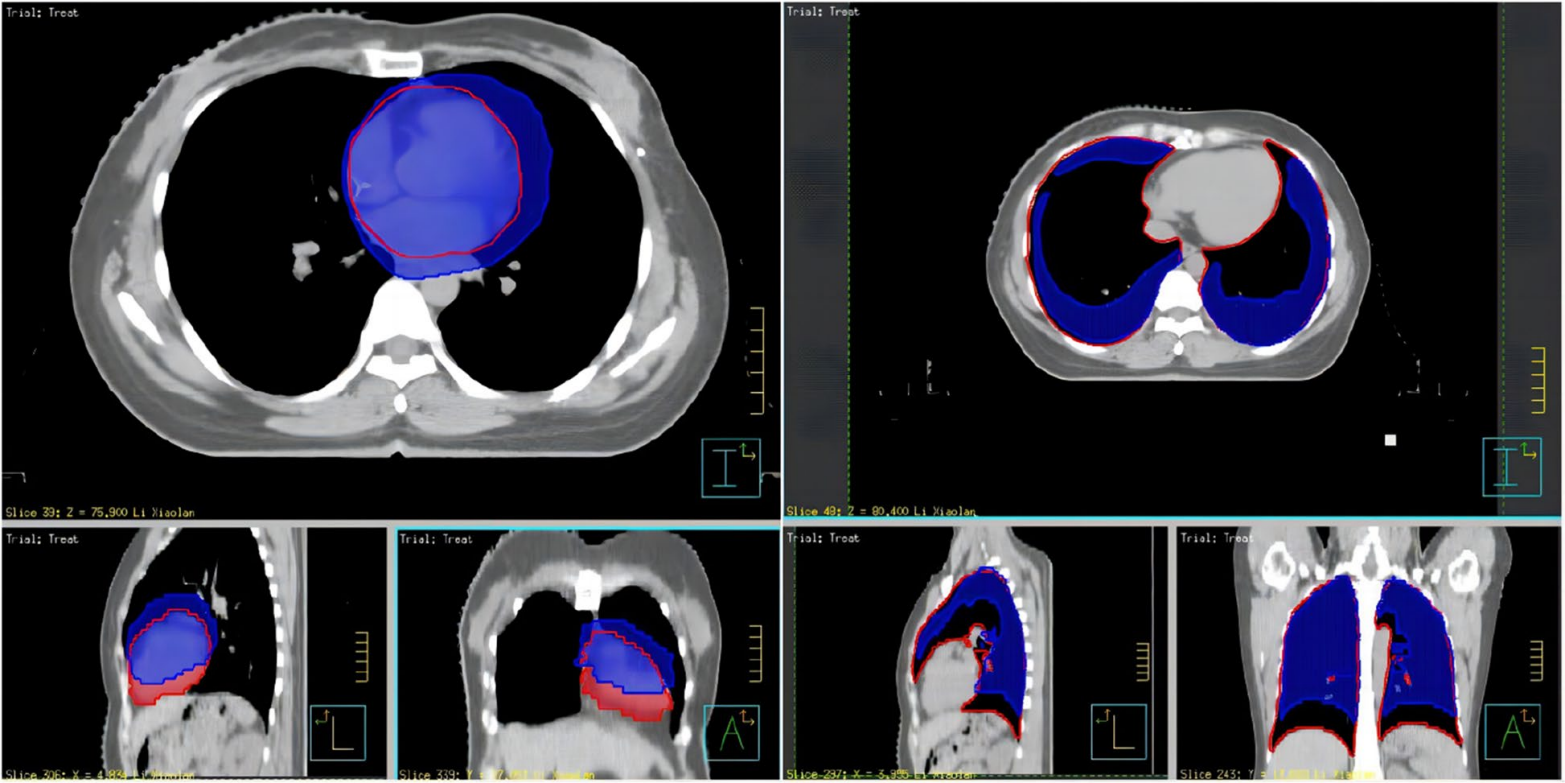
Geometric comparison of OARs (heart and left lung) between FB and aDIBH. Red represented aDIBH and blue represented FB. Free breathing (FB). Abdominal deep inspiration breath-hold (aDIBH)

**Fig. 2:**
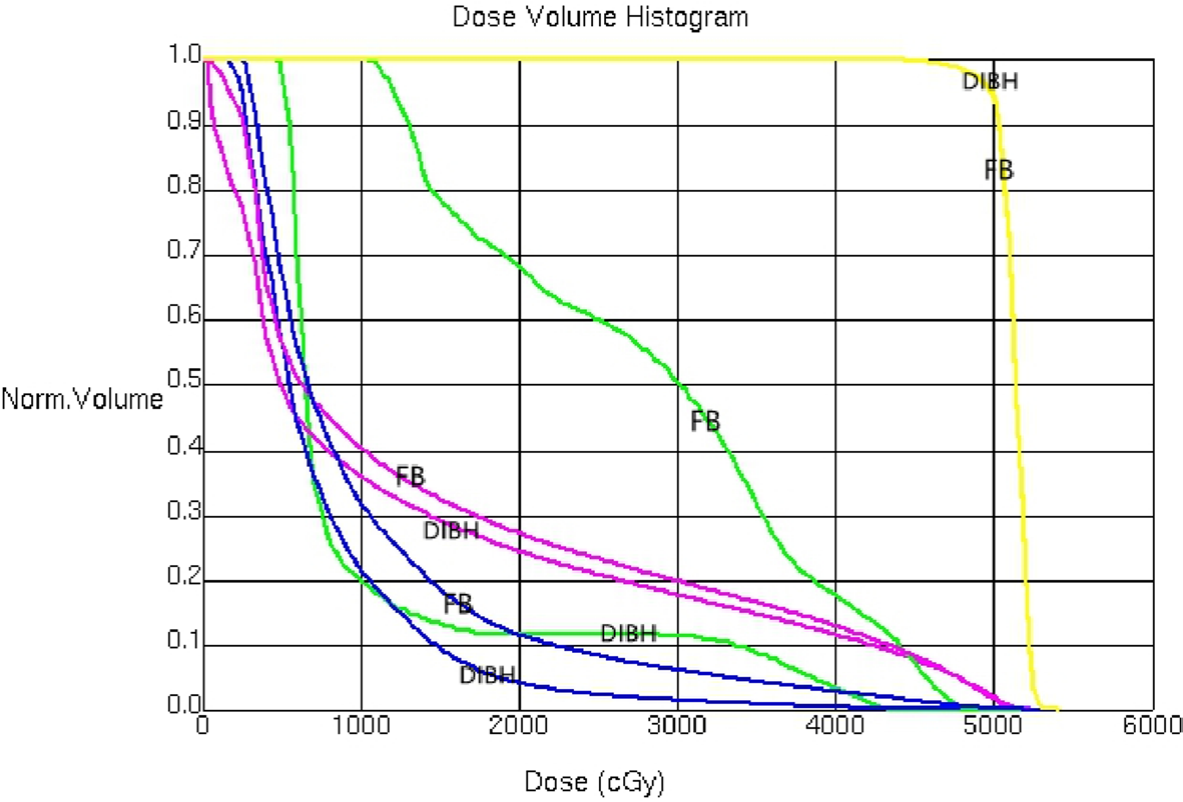
DVH between the PTV and OARs was compared under both FB and aDIBH. Yellow represented PTV, and green represented LADCA, and blue represented heart, and pink represented left lung

Figure 1 showed a geometric comparison of the OARs (heart and left lung) between FB and aDIBH in a typical patient. The same CT image showed two respiratory states: aDIBH and FB. Red represented aDIBH and blue represented FB. Compared with FB, the heart moved farther caudally and away from the chest wall, and the volume of the heart became smaller under aDIBH due to expansion of the lungs. As shown in Fig. 2, the DVH of the PTV and OARs were compared for both the FB and aDIBH. Yellow represented PTV, and green represented LADCA, and blue represented heart, and pink represented left lung. It showed that the radiation dose to the heart, LADCA and left lung under aDIBH was lower than that under FB, and there was no difference in the radiation dose to the PTV between FB and aDIBH.

### Comparison of dosimetry

#### Heart and LAD

**Fig. 3:**
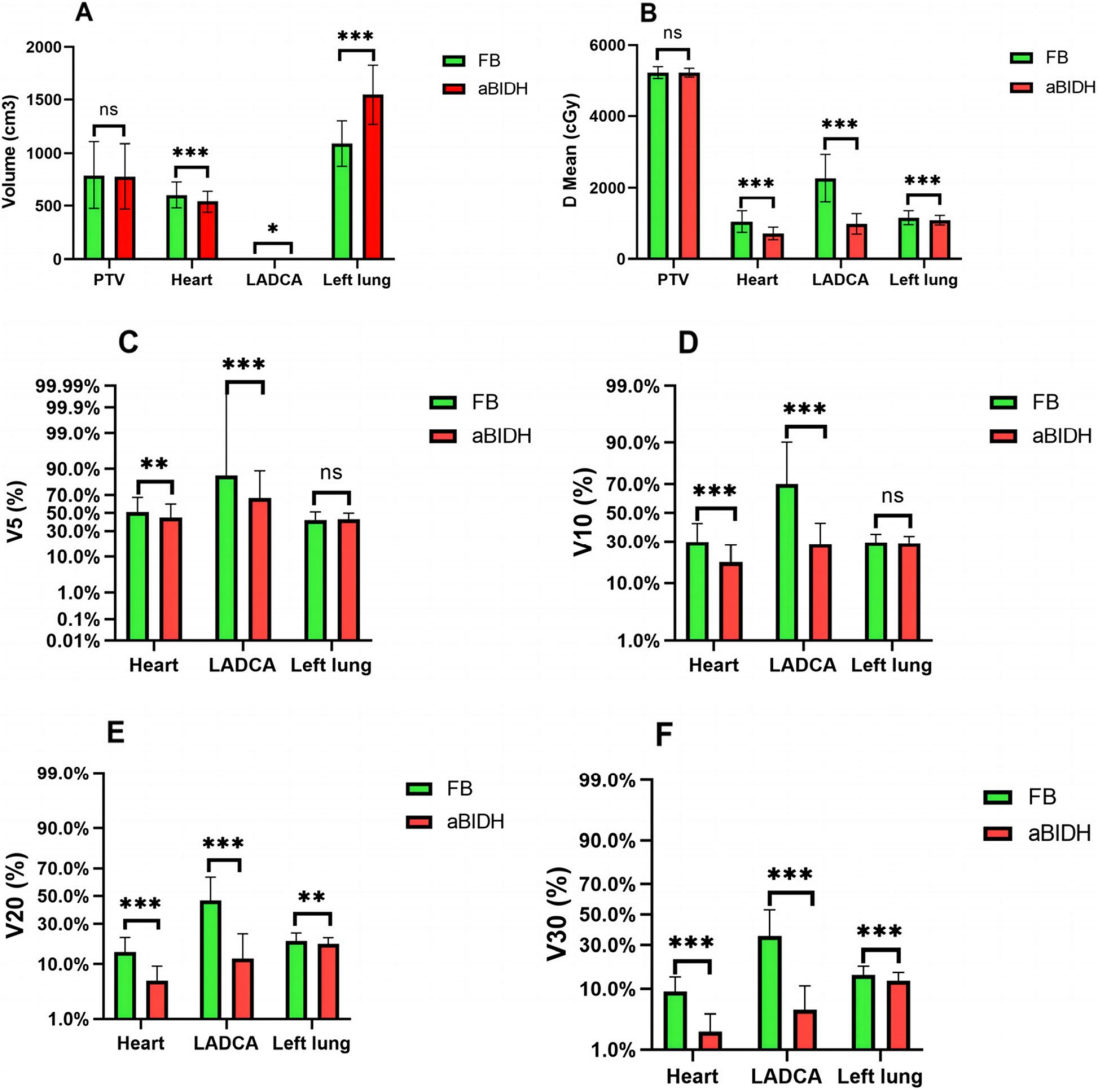
Dosimetric comparison of OARs and PTV on FB and aDIBH. Volume (**A**), D Mean (**B**), V5 (**C**), V10 (**D**), V20 (**E**) and V30 (**F**). FB, free breathing; aDIBH, abdominal deep inspiration breath hold; LADCA, left anterior descending coronary artery; D mean, mean dose. * < 0.05, ** < 0.01, *** < 0.001, ns: no significance

Compared with FB, all parameters of the heart and LADCA under aDIBH were significantly improved (Table 1, Fig. 3). For aDIBH, the volume of the heart and LADCA were respectively reduced by 65.28 ± 54.66 cm3 (*P* < 0.001) and 0.33 ± 0.88 cm3 (*P* < 0.047) (Table 1, Fig. 3A). Meanwhile, the D mean of the heart and LADCA were respectively reduced by 332.79 ± 264.61 cGy (*P* < 0.001) and 1290.37 ± 612.09 cGy (*P* < 0.047) (Table 1, Fig. 3B). V5, V10, V20, and V30 of the heart and LADCA also showed a dosimetric advantage with statistical differences (*P* < 0.05) (Table 1, Fig. 3C-F).

**Table 1:** Dosimetric comparison of OARs and PTV on FB and aDIBH

Parameters	mean ± SD (FB)	mean ± SD (aDIBH)	mean ± SD (Δd)	*P* value
PTV				
Volume (cm^3^)	791.14 ± 314.69	778.45 ± 309.48	-12.70 ± 60.61	0.26
D mean (cGy)	5230.52 ± 165.35	5228.17 ± 122.62	-2.35 ± 93.64	0.89
D max (cGy)	5636.26 ± 465.49	5717.80 ± 464.42	81.53 ± 278.72	0.12
HI	0.09 ± 0.09	1.14 ± 1.13	0.02 ± 0.06	0.11
CI	0.95 ± 0.02	0.95 ± 0.02	0.02 ± 0.41	0.75
Heart				
Volume (cm^3^)	605.79 ± 125.48	540.51 ± 101.39	-65.28 ± 54.66	< 0.001
D Mean (cGy)	1038.74 ± 302.51	705.95 ± 174.16	-332.79 ± 264.61	< 0.001
V5 (%)	51.48 ± 16.01	44.72 ± 15.78	-6.77 ± 14.12	0.01
V10 (%)	29.87 ± 12.49	18.28 ± 9.42	-11.59 ± 12.57	< 0.001
V20 (%)	14.53 ± 7.07	5.49 ± 3.87	-9.05 ± 6.69	< 0.001
V30 (%)	9.23 ± 4.85	2.16 ± 2.09	-7.06 ± 4.82	< 0.001
LADCA				
Volume (cm^3^)	2.81 ± 0.84	2.48 ± 0.77	-0.33 ± 0.88	0.047
D Mean (cGy)	2265.84 ± 671.15	975.47 ± 288.22	-1290.37 ± 612.09	< 0.001
V5 (%)	85.97 ± 18.28	66.87 ± 22.05	-19.09 ± 20.05	< 0.001
V10 (%)	69.95 ± 20.19	28.34 ± 14.14	-41.61 ± 22.07	< 0.001
V20 (%)	46.65 ± 17.41	11.98 ± 11.75	-34.67 ± 17.02	< 0.001
V30 (%)	35.47 ± 17.76	4.88 ± 6.03	-30.58 ± 16.99	< 0.001
Left lung				
Volume (cm^3^)	1089.33 ± 212.28	1547.69 ± 280.93	458.36 ± 197.16	< 0.001
D Mean (cGy)	1147.47 ± 196.59	1077.54 ± 135.81	-69.94 ± 117.73	< 0.001
V5 (%)	41.91 ± 9.56	43.10 ± 6.76	1.19 ± 9.01	0.48
V10 (%)	29.48 ± 5.31	29.03 ± 4.33	-0.45 ± 3.12	0.43
V20 (%)	19.70 ± 4.56	18.24 ± 3.26	-1.46 ± 2.65	0.01
V30 (%)	14.85 ± 3.83	12.59 ± 3.33	-2.26 ± 3.07	< 0.001

*FB* Free breathing, *aDIBH* Abdominal deep inspiration breath-hold, *LADCA* Left anterior descending coronary artery, *SD* Standard deviation, *D mean* Mean dose, *D max* Maximum dose, *HI* Homogeneity index, *CI* Conformity index, *mean* ± *SD (Δd)* The difference between mean ± SD(DIBH) and mean ± SD(FB)

#### Left lung

Compared with FB, the volume of the left lung under aDIBH was increased by 458.36 ± 197.16 cm3 (*P* < 0.001) (Table 1, Fig. 3A), and the D mean was reduced by 69.94 ± 117.73 cGy (*P* < 0.001) (Table 1, Fig. 3B). Meanwhile, V20 and V30 of the left lung also showed a dosimetric advantage with statistical differences (*P* < 0.05) (Table 1, Fig. 3E-F).

#### PTV

Compared with FB, the volume of the PTV under aDIBH were respectively 778.45 ± 309.48 cm3 and 791.14 ± 314.69 cm3 with no statistical differences (*P* = 0.26) (Table 1, Fig. 3A), and there was no significant difference in other parameters (D mean, D max, HI, and CI) between the two groups (*P* > 0.05).

## Discussion

Results of a large meta-analysis by the Early Breast cancer Trialists’ Group showed that postoperative radiotherapy could significantly improve the survival rate and decrease the local recurrence rate [14]. However, postoperative radiotherapy could lead to early and late side effects of OARs, especially in the heart, anterior cardiac structures and ipsilateral lung.

In recent years, DIBH technology has been widely used in patients with breast cancer to reduce the radiation dose of OARs [15, 16]. Different breath-holding methods have been utilized for DIBH. The two dominant methods were ABC system (Elekta, Stockholm, Sweden) and RPM system (Varian Medical Systems, Palo Alto, CA) [8]. In recent years, OSMS has become increasingly advanced tools (AlignRT, Vision RT Ltd., London, UK; Sentinel, C-RAD, Uppsala, Sweden) [9]. In addition, aDIBH had more advantages in reducing the irradiation dose of OARs than tDIBH [7]. In our study, we reported the experience of our single institution with OARs-sparing radiotherapy with aDIBH using an audio-guided device.

Firstly, radiation-related heart disease (RRHD) was the most serious adverse reaction to radiotherapy for breast cancer. Radiotherapy could cause small vessel microvascular and coronary artery macrovascular disease, which could lead to myocardial fibrosis, coronary artery disease, and ultimately ischemic heart disease [17–19]. There was a dose–response relationship between the cardiac exposure dose and the incidence of RRHD. Evidence existed that any reduction in radiation exposure to the heart would lower the incidence of ischemic heart disease in patients with breast cancer. A previous study [6] showed that mean cardiac dose of 300 cGy (1000 cGy) increased the risk of death from ischemic heart disease from 1.9% to 2.4% (1.9% to 3.4%) and the risk of at least one acute coronary event from 4.5% to 5.4% (1.9% to 7.4%) in patients with no preexisting cardiac risk factors. In addition, incidental exposure of the heart to radiotherapy for breast cancer would increase the rate of major coronary events by 7.4% per gray. In our study, due to the expansion of the lungs, location of heart was moved farther caudally and away from chest wall, and the volume of heart becomed smaller under the aDIBH (540.51 ± 101.39 cm3 and 605.79 ± 125.48 cm3). Because the above changes, the aDIBH could lead to lower radiation dose of heart and LADCA (D mean, V5, V10, V20 and V30), in which the D mean of the heart and LADCA were respectively reduced by 332.79 ± 264.61 cGy and 1290.37 ± 612.09 cGy. Meanwhile, the reduction on the radiation dose of LADCA was more significant than that in the heart (Fig. 2, Fig. 3). This meant that aDIBH could effectively reduce the incidence rate of RRHD, especially the occurrence of coronary artery disease.

Secondly, radiation pneumonitis (RP) was an acute manifestation of radiation-induced lung injury and one of the main dose-limiting toxicities in patients receiving thoracic radiation therapy. There was also a dose–response relationship between the pulmonary exposure dose and the incidence rate of RP. It mainly depended on the volume of the irradiated lung [20]. D mean and V20 were often associated with RP and were most commonly used in clinical practice, and other variables (V5, V10, and V30) were also predictive [21]. The dose-volume limit of the ipsilateral lung (V20 ≤ 30%) could reduce the incidence of RP and short-term changes in lung function [22]. In our study, compared with the FB, the volume of the left lung under the aDIBH increased by 458.36 ± 197.16 cm3, and the increase in pulmonary volume meant a decrease in radiation dose (D mean, V20, and V30), which in turn reduced the incidence rate of RP. Meanwhile, radiation therapy under aDIBH did not lead to a loss of dose for the PTV (D mean, D max) and target coverage (CI, HI).

Compared with previous studies, the dose of OARs in our study was higher. The reasons might be as follows: on the one hand, it might be due to the different of radiation field arrangements, such as the study of Sung K et al. [23], treatment plans were generated by the treatment planning system with a pair of wedged tangential fields, however six to eight main tangential conformal fields were used with the field-in-field technique in our study. On the other hand, all treatment plans had to meet the criterion that at least 95% of the PTV received 100% of the prescribed dosage in our study, which might have led to an increased radiation dose of OARs. However, in the other study [24], planning aims were to cover ≥ 95% of the PTV with ≥ 95% of the prescribed dose. Additionally, radiotherapy technologies also affect the dose of OARs. Among the three radiotherapy technologies (3D-CRT, IMRT, and VMAT), the D mean of the heart was lowest in aDIBH IMRT and 130 cGy lower than in aDIBH VMAT (*P* = 0.002), so aDIBH IMRT resulted in the best heart-sparing effect [25].

Finally, some problems about DIBH technique need to be explored. Regarding the selection criteria to predict which patients would benefit most from the DIBH technique other than left breast laterality, a strong linear correlation was found between the maximum heart distance (MHD) and the mean heart dose. For every 1 cm increase in MHD, the mean heart dose increased by 2.9% on average (95% CI: 2.5–3.3) [26]. In addition, parasagittal cardiac contact distance (CCD) was a potentially good predictor of cardiac exposure: the longer the CCD, the higher the dose, and at least 75% of patients with left-sided breast cancer might benefit from the DIBH technique in terms of potentially clinically relevant dose reduction to cardiac structures [27]. For selected patients with unfavorable cardiac anatomy, defined as having > 10 cm^3^ of the heart receiving 50% of the prescribed dose (V50% > 10 cm^3^) on the free-breathing automated treatment plan, the DIBH technique could significantly reduce the dose to the LADCA and heart, potentially reducing cardiac risk [28].

There was still controversy about whether to perform prophylactic irradiation on the ipsilateral internal mammary chain (IMC) because of conflicting data on the benefits and losses of this treatment strategy [29, 30]. In a study about aDIBH in postoperative adjuvant radiotherapy for left breast cancer with IMC coverage compared with FB, even if IMC was included in CTV, the radiation dose to the heart was reasonable low [21].

Our study has some limitations. The cohort size of 35 patients is modest. The patient comfort and treatment times are not recorded. Respiratory training and cooperation of patients are crucial for aDIBH, otherwise it may lead to a use of invalidated audio guided device and in actuality OARs may have received more dose as compared to as seen on planning scan.

## Conclusions

In conclusion, postoperative radiotherapy using aDIBH technology after breast-conserving surgery in patients with left breast cancer reduced the irradiation dose to the heart, LADCA and left lung without compromising the target coverage compared with FB. All patients who compied with the requirements completed their treatment sessions with aDIBH. For some hospitals without monitoring equipments (such as ABC and RPM) for performing aDIBH, aDIBH using an audio-guided device under active breathing training is easily feasible in daily practice and significantly reduces the doses of the OARs and may be used in the clinic. However, this requires further comparative clinical data.

## Data Availability

The data that support the findings of this study were available from the first author and corresponding author upon reasonable request.

## Abbreviations

DIBH: Deep inspiration breath-hold
aDIBH: Abdominal deep inspiration breath hold
tDIBH: Thoracic deep inspiration breath hold
vDIBH: Voluntary deep inspiration breath hold
FB: Free breathing
ABC: Active breathing coordinator
RPM: Real-time position management
OSMS: Optical surface management system
LADCA: Left anterior descending coronary artery
OARs: Organs at risk
CTV: Clinical target volume
PTV: Planning target volume
IARC: International Agency for Research on carcinoma
RIHD: Radiation-induced heart disease
RP: Radiation pneumonitis
3D-CRT: Three-dimensional conformal radiotherapy
IMRT: Intensity-modulated radiotherapy
VMRT: Volume of rotating intensity modulated radiotherapy
CT: Computed tomography
RTOG: Radiation Therapy Oncology Group
HI: Homogeneity index
CI: Conformity index
SD: Standard deviation
DVH: Dose-volume histogram
AJCC: American Joint Committee on Cancer
IMC: Internal mammary chain
